# Clinical prediction rules for childhood urinary tract infections: a cross-sectional study in ambulatory care

**DOI:** 10.3399/BJGPO.2021.0171

**Published:** 2022-04-06

**Authors:** Hanne Ann Boon, Jan Y Verbakel, Tine De Burghgraeve, Ann Van den Bruel

**Affiliations:** 1 EPI-Centre, Department of Public Health and Primary Care, KU Leuven, Leuven, Belgium; 2 Nuffield Department of Primary Care Health Sciences, University of Oxford, Oxford, UK

**Keywords:** ambulatory care, child, clinical decision rules, primary health care, urinary tract infections, validation studies (publication type)

## Abstract

**Background:**

Diagnosing childhood urinary tract infections (UTIs) is challenging. Clinical prediction rules may help to identify children that require urine sampling. However, there is a lack of research to determine the accuracy of the scores in general practice.

**Aim:**

To validate clinical prediction rules (UTI Calculator [UTICalc], A Diagnosis of Urinary Tract Infection in Young Children [DUTY], and Gorelick score) for paediatric UTIs in primary care.

**Design & setting:**

Post-hoc analysis of a cross-sectional study in 39 general practices and two emergency departments (EDs). The study took place in Belgium from March 2019–March 2020.

**Method:**

Physicians recruited acutely ill children aged ≤18 years and sampled urine systematically for culture. Per rule, an apparent validation was performed, and sensitivities and specificities were calculated with 95% confidence intervals (CIs) per threshold in the target group. For the DUTY coefficient-based algorithm, a logistic calibration was performed and the area under the receiver operating characteristic curve (AUC) was calculated with 95% CI.

**Results:**

Of 834 children aged ≤18 years recruited, there were 297 children aged <5 years. The UTICalc and Gorelick score had high-to-moderate sensitivity and low specificity: UTICalc (≥2%) 75% and 16%, respectively; Gorelick (≥2 variables) 91% and 8%, respectively. In contrast, the DUTY score ≥5 points had low sensitivity (8%) but high specificity (99%). Urine samples would be obtained in 72% versus 38% (UTICalc), 92% versus 38% (Gorelick) or 1% versus 32% (DUTY) of children, compared with routine care. The number of missed infections per score was 1/4 (UTICalc), 2/23 (Gorelick), and 24/26 (DUTY). The UTICalc + dipstick model had high sensitivity and specificity (100% and 91%), resulting in no missed cases and 59% (95% CI = 49% to 68%) of antibiotics prescribed inappropriately.

**Conclusion:**

In this study, the UTICalc and Gorelick score were useful for ruling out UTI, but resulted in high urine sampling rates. The DUTY score had low sensitivity, meaning that 92% of UTIs would be missed.

## How this fits in

Three clinical prediction rules for UTIs in children were previously developed to identify children requiring urine sampling. These prediction rules have not yet been validated in another setting and, therefore, the robustness of these scores has not yet been established. In this dataset, the UTICalc and Gorelick score had low specificity, leading to a high number of children in whom a urine sample would have to be obtained (72% and 92%) compared with standard clinical care (38%). The DUTY score had a low sensitivity, meaning that 92% of UTIs would be missed.

## Introduction

UTIs occur in 3%–14% of acutely ill children in ambulatory care.^1^ Diagnosis is important because early antibiotic treatment can prevent progression to a severe illness and might prevent renal scarring.^2^

Ruling out UTIs is challenging because children with UTI have non-specific clinical features.^3^ Additionally, guidelines are not very specific in describing which children require sampling.^4–6^

Urine sampling in all children is undesirable, as urine collection is difficult to combine with routine care, particularly in young children, and not cost-effective.^7–9^ Prediction rules may help to identify children who require urine sampling. Since missing a UTI is more problematic than oversampling, any clinical prediction rule should have high sensitivity because this minimises the number of false negatives. The target population for a prediction rule should ideally reflect the population seen in daily practice, which is a broad spectrum of acutely ill children.

In a recent systematic review,^10^ three prediction rules for UTI were identified, all based on clinical features:^11–13^

1. The DUTY score is a points-based algorithm, derived from a large cohort study (*n* = 7163) in general practices in the UK, including acutely ill children aged <5 years. The score was validated internally through bootstrapping (area under the receiver operating characteristic curve analysis [AUC] 0.89 [95% CI = 0.85 to 0.95]).^11^
2. UTICalc was derived using a nested case-control study in the US with internal validation using a separate sample (AUC 0.81 [95% CI = 0.72 to 0.89]) of febrile children aged <2 years evaluated for UTI at the ED (*n* = 2070). The calculator is available online: https://uticalc.pitt.edu.^12^
3. Gorelick *et al* ^13^ derived a prediction rule using a prospective cohort study in febrile girls aged <2 years (*n* = 1469) at the ED (AUC 0.76), with internal validation using a case-control study (AUC 0.72).^14^ This score is implemented in the American Academy of Pediatrics (AAP) guidelines.^15^

To the authors’ knowledge, these prediction rules have not yet been validated externally and, therefore, the robustness of these scores has not yet been established. The population of the UTICalc and Gorelick score consisted of children at higher risk of UTI, and, therefore, might be less applicable to general practice.

The aim of this study was to validate clinical prediction rules for UTI in primary care in order to determine the accuracy of these scores.

## Method

### Study registration

This was a post-hoc analysis of the ERNIE4 study, of which the methods and results are reported elsewhere.^16^ The ERNIE4 study is reported following the Standards for Reporting of Diagnostic Accuracy Studies (STARD) 2015 guidelines.^16^

### Study design

The ERNIE4 study was a multicentre, prospective cross-sectional study in 39 general practices and two EDs in Belgium (March 2019–March 2020). Urine was sampled systematically and sent for analyses to one of four laboratories (AML, CMA, Antwerp; AZ Maria Middelares, Ghent; and Jessa Ziekenhuis, Hasselt). For toilet-trained children, samples were obtained by midstream voiding at the time of study inclusion. For non-toilet trained children, physicians were asked to perform the Quick-Wee method, that is, a direct catch of a first-stream sample;^17^ if unsuccessful, urine was collected using adhesive bags. In such a case, parents were asked to provide the sample within 24 hours after inclusion.

### Participants

Children aged between 3 months and 18 years with an acute illness ≤10 days duration were eligible. Patients were not included if they presented with a traumatic injury, had a urinary catheter, were critically unstable, were referred to the hospital, or had been on immunosuppressive medication (≤30 days) or antibiotics (≤7 days).

### Clinical prediction rules

Prediction rules for UTI in children were selected based on clinical features, to determine which children required urine sampling. For each rule, those patients from the ERNIE4 study were selected based on the inclusion criteria of the rules’ derivation studies and the urine culture threshold was adapted accordingly, for optimal between-study comparison. The prediction rule variables are presented in Table 1 and a comparison with the ERNIE4 variables are provided in Supplementary Table S1.

**Table 1.** Diagnostic accuracies of clinical prediction rules for childhood urinary tract infections

Category	UTICalc (≥2%)	Gorelick score (≥2 variables)	DUTY (≥5 points) points-based algorithm
Number of UTIs / number of children included, *n*	4 / 96	23 / 100	26 / 297
Population	3 months–2 years with fever and no urinary tract abnormalities	3 months–2 years with fever	3 months–5 years with acute illness
Variables	Age <12 months; fever ≥39°C; non-Black ethnicity; female sex; uncircumcised male; and fever without source^a^	Aged <12 months; White ethnicity; fever ≥39°C; fever ≥2 days; fever without source^b^	Dysuria (2 points); malodorous urine (2 points); history of UTI (1 point); absence of severe cough (2 points); severity of illness (2 points when >6 on a scale of 0–10)
Reference standard	1 or 2 pathogens >5 × 10^4^ CFU/ml and pyuria	1 or 2 pathogens >5 × 10^4^ CFU/ml	1 pathogen >10^5^ CFU/ml
Sensitivity / specificity, derivation study, % (95% CI)	95 (NR) / 35 (NR)	95 (85 to 99) / 31 (28 to 34)	52 (39 to 64) / 95 (94 to 95)
Sensitivity / specificity, ERNIE4 study, % (95% CI)	75 (19 to 99) / 16 (9 to 25)	91 (72 to 99) / 8 (3 to 16)	8 (1 to 25) / 99 (96 to 100)
Urine sampling rate followingprediction rule / routine care, % (95% CI)	72 (62 to 81) / 38 (28 to 48)	92 (85 to 97) / 38 (29 to 48)	1 (0.8 to 4) / 32 (26 to 37)
Number of missed infections,prediction rule versus routine care, *n*	1/4 versus 2/4	2/23 versus 17/23	24/26 versus 16/26

^a^Defined in the original study as no upper respiratory tract infection, no bronchiolitis, no pneumonia, no acute otitis media, no gastroenteritis, no meningitis, and no viral syndrome. ^b^defined in the original study as discharge diagnoses: ‘fever’, ‘fever without source’, or ‘viral infection’. CFU = colony-forming unit. DUTY = Diagnosis of Urinary Tract Infections in Children. NR = not reported. UTI = urinary tract infection. UTICalc = UTI Calculator.

1. For the DUTY models, all children aged <5 years were selected. The authors derived a coefficient-based algorithm and a points-based algorithm (see Supplementary Table S1). Urine sampling is recommended for children scoring ≥5 points on the points-based model. Dipstick test results can be added to decide on initiation of antibiotic treatment but the optimal threshold is unclear as none of the thresholds were cost-effective in the original study. In this study, ≥6 points was used as threshold for the DUTY score + dipstick model, meaning that treatment would be initiated when the clinical model was positive and either blood, nitrite, or leukocyte esterase (LE) were positive, in order to obtain a high sensitivity.
2. For UTICalc (version 3.0), all febrile children (≥38°C) aged <2 years without urinary tract abnormalities were selected. After urine had been obtained (UTI probability ≥2%), dipstick or microscopy results (hemocytometer model) were added to the score to guide initiation of treatment (probability ≥5%).
3. For the score by Gorelick *et al*, all febrile children aged <2 years were selected. In contrary to the original study, both girls and boys were included, because UTIs occur frequently in boys aged <1 year, and implementation of a score for only girls did not seem practical.

### Reference standard

In the study, UTI was defined as a single pathogen ≥10^5^ colony-forming units per millilitre (CFU/ml) on urine culture.^6^ Contamination was defined as multiple pathogens or one pathogen <10^5^ CFU/ml. Samples were excluded if there was no result for culture or if the sample was received >72 hours after inclusion in the laboratory.

For the DUTY models, the reference standard was one pathogen ≥10^5^ CFU/ml; for the Gorelick score, a pathogen ≥5 × 10^4^ CFU/ml; and for the UTICalc, a pathogen ≥5 × 10^4^ CFU/ml with pyuria; for example, LE ≥trace or white blood cells (WBC) (≥5/high-power field or ≥10/microliter [µl]).

### Data collection

At inclusion, clinical features were recorded for each child by the treating physician. Additionally, 30-day follow-up information was collected including laboratory or imaging results and hospital records, which were all conducted as part of routine care and not study-specific. The treating physician was asked to formulate a working hypothesis at the end of the initial consultation. In the analyses, suspicion of UTI was defined as a working hypothesis: ‘UTI’, ‘cystitis’, or ‘pyelonephritis‘.

All children underwent study-specific urine sampling. For each child, a study-specific urine culture was performed by laboratory technicians that were blinded to the index tests. Additionally, physicians were blinded for all study-specific test results, and, therefore, they were instructed to obtain an additional urine sample for clinical management if they deemed it necessary.

### Statistical analyses

All statistical analyses were performed using R (version 4.0.4). Sensitivities, specificities, and positive and negative likelihood ratios were calculated with 95% CI for clinical features (‘epiR’ package).^18^ When values for clinical features were missing, they were considered as normal.

For the DUTY models, an apparent validation (points-based model) and a logistic regression (coefficient-based model) were performed.^19^ The AUC was calculated with 95% CI (‘pROC’ package).^20^ A calibration plot was made using the ‘val.prob.ci.2’ function (‘CalibrationCurves’ package).^21^ For the UTICalc and Gorelick score, the original regression coefficients were not available and, therefore, an apparent validation was performed, that is, the model performance was assessed as is, without modifications.

As sensitivity analyses, urine culture thresholds were adapted to: one pathogen of ≥10^5^ CFU/ml following the European Association of Urology guidelines^6^ and lowered the threshold to 5 × 10^4^ CFU/ml with pyuria (LE ≥trace or ≥10WBC/µl), following the AAP guidelines.^4^

## Results

### Study recruitment

**Figure 1.**
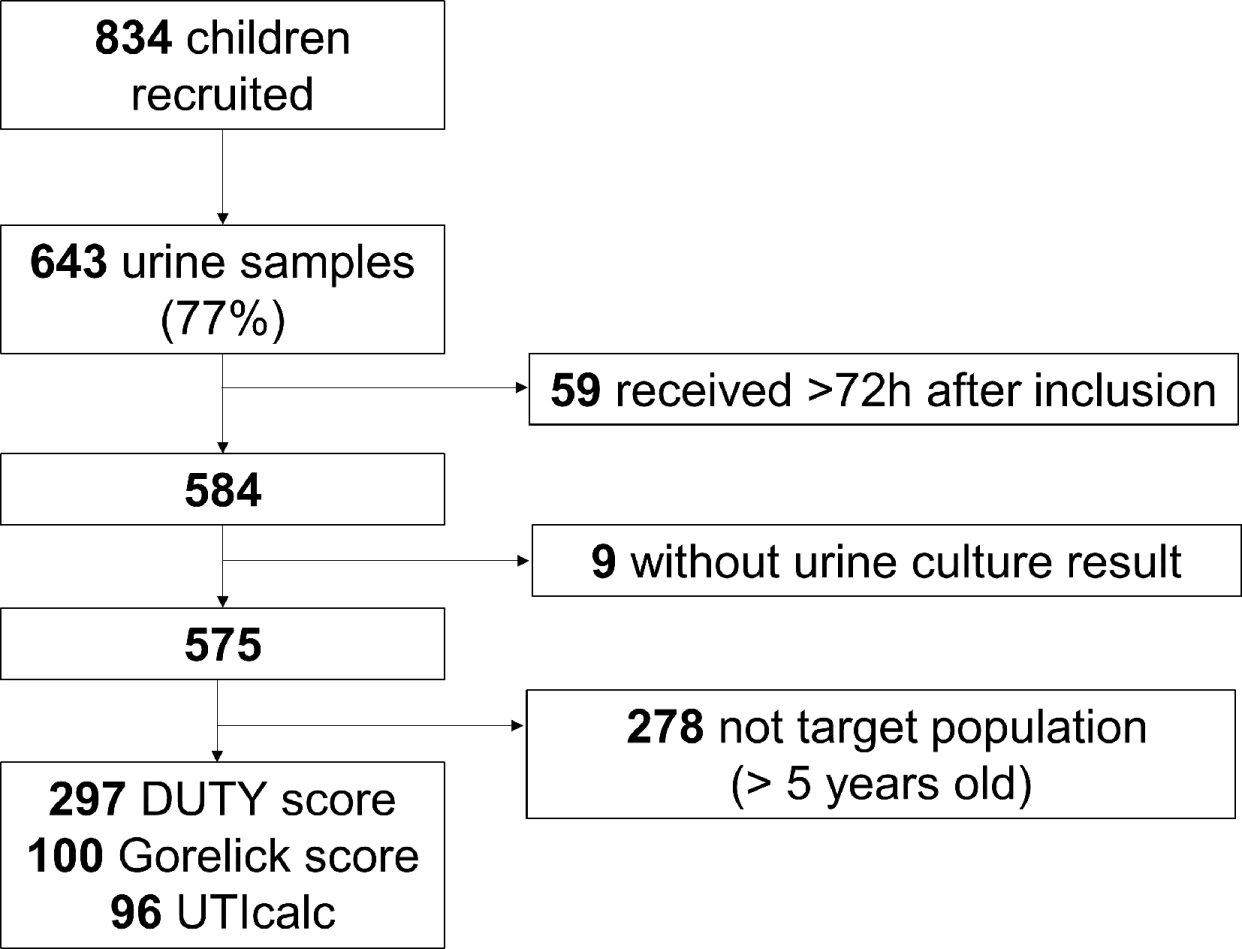
Flowchart of recruited children and urine samples obtained. DUTY = Diagnosis of Urinary Tract Infections in Children. h = hours. UTICalc = UTI Calculator.

There were 834 children recruited, of whom 643 children provided a urine sample. After exclusion of 68 samples because of arrival >72 hours after inclusion or no results for culture, 575 urine samples were available for analysis, of which 297 samples were from children aged <5 years (Figure 1). The median number of recruited children per practice was 13 (range 1–87).

### Patient characteristics

Patient characteristics are listed in Table 2 per subgroup. The median age was 6 years (IQR 4–10) and 48% were girls. There were 51 children (9%) with a previous history of UTI of whom nine had vesicoureteral reflux. Most children presented with respiratory (81%) or abdominal features(34%), while 8% presented with either frequency, dysuria, or malodorous urine.

**Table 2.** Characteristics of acutely ill children

Characteristic	Aged ≤18 years, *n* = **575**	DUTY sample, *n* = **297**	Gorelick sample, *n* = **100**	UTICalc sample, *n* = **96**
Age, years, median (IQR)	6.38 (3.97–10.25)	2.60 (1.12–3.75)	0.94 (0.56–1.49)	0.94 (0.56–1.49)
**Sex, *n* (%)**				
Girl	276 (48)	134 (45)	38 (38)	37 (39)
Boy	298 (52)	163 (55)	62 (62)	59 (61)
Missing	1 (0.2)	0 (0)	0 (0)	0 (0)
Circumcised boys, *n* (% of boys)	31 (10)	12 (7)	5 (8)	5 (8)
Fever: 'yes', *n* (%)	415 (72)	249 (84)	100 (100)	96 (100)
Duration of illness, days, median (IQR)	3.00 (1.00–4.00)	2.00 (1.00–3.00)	2.00 (1.00–3.00)	2.00 (1.00–3.00)
**Dysuria, *n* (%)**				
Yes	31 (5)	11 (4)	1 (1)	1 (1)
No	535 (93)	282 (95)	99 (99)	95 (99)
Missing	9 (2)	4 (1)	0 (0)	0 (0)
**Frequency, *n* (%)**				
Yes	26 (5)	9 (3)	1 (1)	1 (1)
No	539 (94)	283 (95)	99 (99)	95 (99)
Missing	10 (2)	5 (2)	0 (0)	0 (0)
**Malodorous urine, *n* (%)**				
Yes	16 (3)	9 (3)	3 (3)	3 (3)
No	547 (95)	284 (96)	97 (97)	93 (97)
Missing	12 (2)	4 (1)	0 (0)	0 (0)
**Abdominal pain, *n* (%)**				
Yes	121 (21)	41 (14)	5 (5)	5 (5)
No	450 (78)	253 (85)	95 (95)	91 (95)
Missing	4 (1)	3 (1)	0 (0)	0 (0)
**History of UTI, *n* (%)**				
Yes	51 (9)	23 (8)	10 (10)	9 (9)
No	514 (89)	270 (91)	90 (90)	87 (91)
Missing	10 (2)	4 (1)	0 (0)	0 (0)
**VUR, *n* (%)**				
Yes	10 (2)	5 (2)	2 (2)	2 (2)
No	555 (97)	288 (97)	98 (98)	94 (98)
Missing	10 (2)	4 (1)	0 (0)	0 (0)

DUTY = Diagnosis of Urinary Tract Infections in Children. IQR = interquartile range. UTI = urinary tract infection. UTICalc = UTI Calculator. VUR = vesicoureteral reflux.

In addition to the study-specific urine sample that was obtained in all children, treating physicians requested a urine sample for clinical management in 151/575 children (26%). The sensitivity and specificity of a UTI working hypothesis was 7% (95% CI = 2% to 20%) and 95% (95% CI = 93% to 97%), respectively.

### Samples and UTI prevalence

For the DUTY models, there were 297 children aged <5 years, of which 26 (9%) had a UTI (one pathogen ≥10^5^ CFU/ml) (Table 1). For the UTICalc, there were 96 febrile children aged <2 years and four of them (4%) had a UTI (pathogen ≥5 × 10^4^ CFU/ml with pyuria). For the Gorelick, there were 100 febrile children aged <2 years of which 23 (23%) had a UTI (pathogen ≥5 × 10^4^ CFU/ml).

Of all children with UTI recruited in the ERNIE4 study, two children were hospitalised with pyelonephritis. Both children had elevated C-reactive protein levels (171 and 170 mg/L) at the general practice.

### Obtaining urine samples

**Figure 2.**
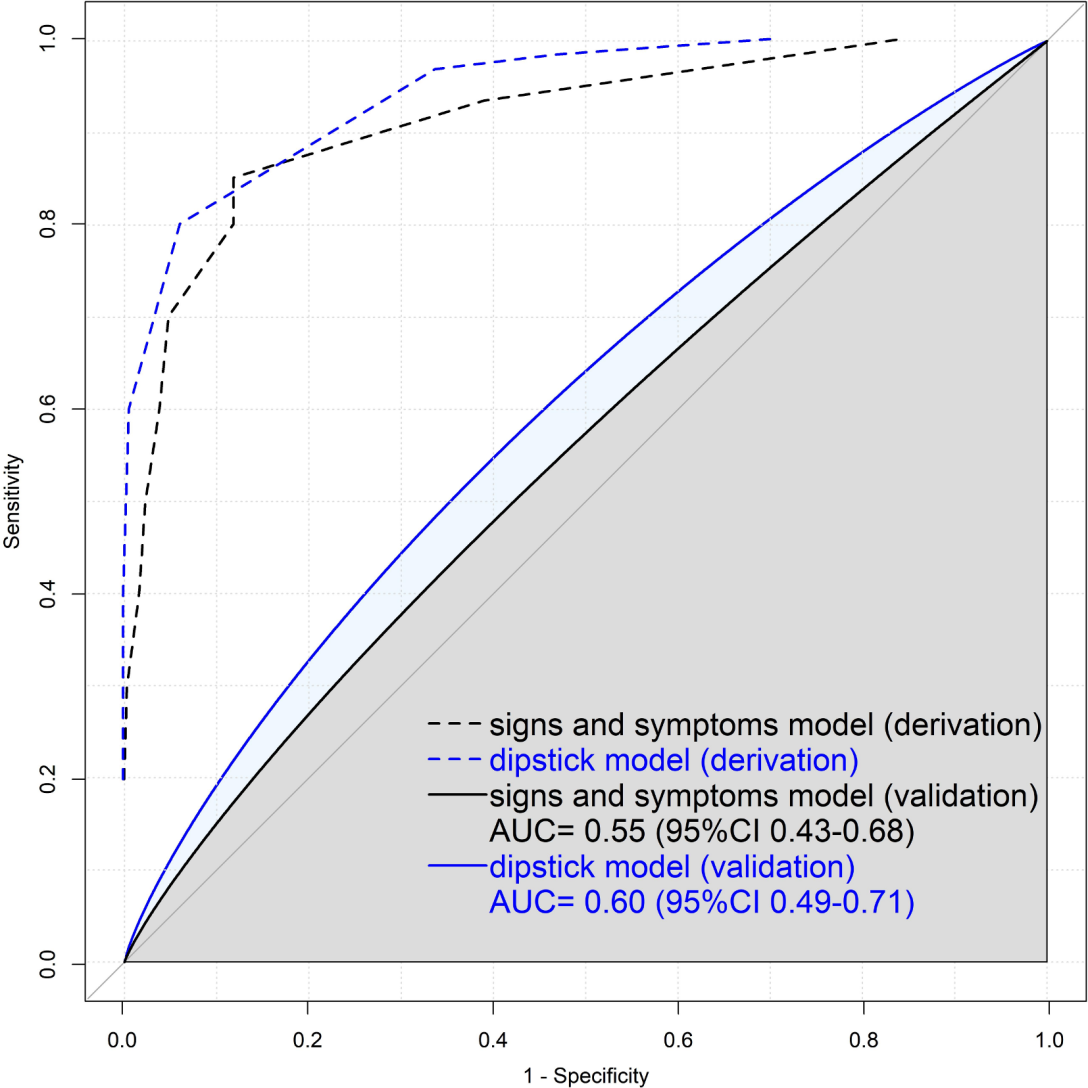
Receiver operating characteristic (ROC) plot of the DUTY coefficient-based model for urinary tract infection in children aged <5 years. ROC plot showing sensitivity versus 1-specificity. The recalibrated intercept and slope were –1.5731 and 0.1473 for the signs and symptoms coefficient-based model and –0.9252 and 0.2526 for the dipstick coefficient-based model, respectively. The calibration was weak for both scores (see Supplementary Figure S1). AUC = area under the ROC curve.

The diagnostic accuracies of the prediction rules are presented in Table 1, and Figures 2 3.

**Figure 3.**
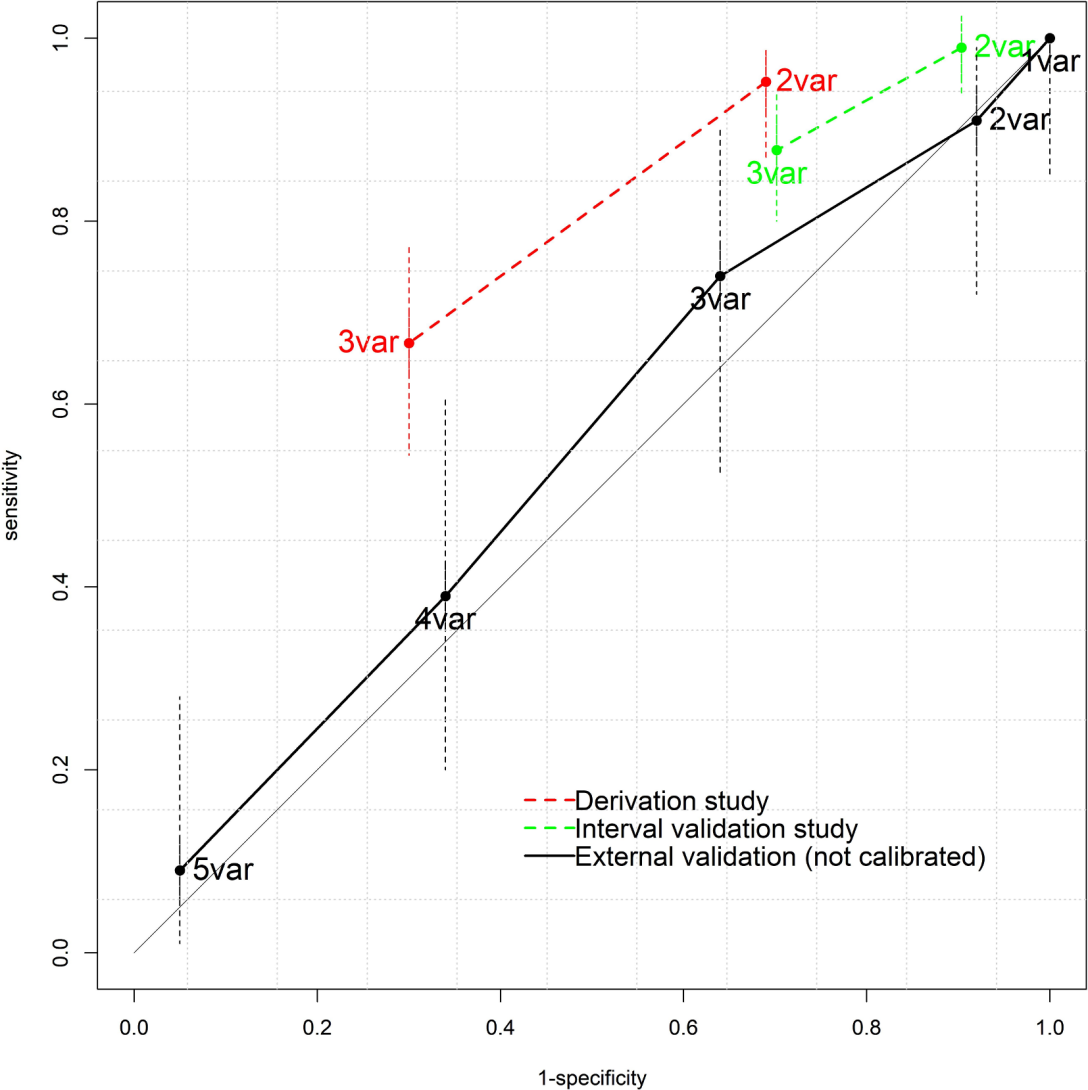
Receiver operating characteristic (ROC) plot of the Gorelick score for urinary tract infection in children aged <2 years. ROC plot showing sensitivity versus 1-specificity. var = variable.

The UTICalc (≥2%) and Gorelick score (≥2 variables) had high-to-moderate sensitivity and low specificity (Table 1). In contrast, the DUTY score (≥5 points) had low sensitivity but high specificity. Assuming a urine sample would be requested in children testing positive on the prediction rule, the urine sampling rate would be 92% (Gorelick), 72% (UTICalc), and 1% (DUTY), compared with 38%, 38%, and 32% for standard care.

**Figure 4.**
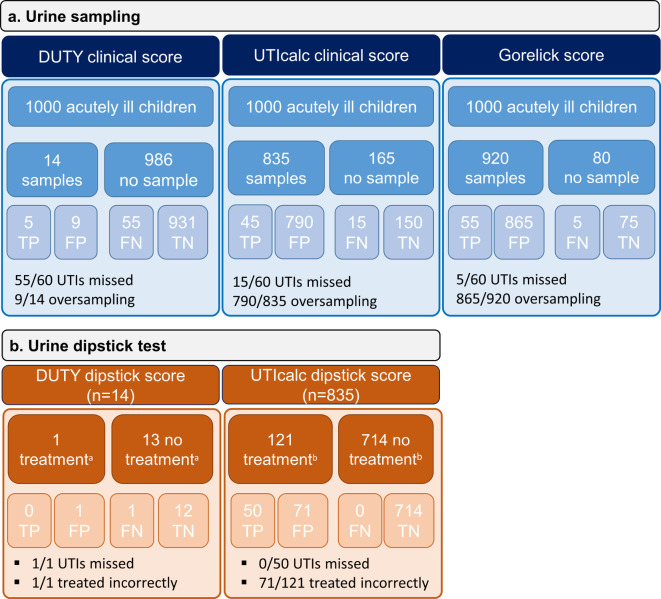
Simulation for 1000 acutely ill children based on sampling and treatment strategies. Simulation for 1000 acutely ill children based on ERNIE4 study results (assuming UTI prevalence of 6%).^22^ ^a^Positive DUTY dipstick model ≥6 points (for example, clinical model positive and ≥1 variable on the dipstick test positive). ^b^Positive UTICalc dipstick model (probability of UTI ≥5% in children with positive clinical model). DUTY = Diagnosis of Urinary Tract Infections in Children. FN = false negatives. FP = false positives. TN = true negatives. TP = true positives. UTICalc = UTI Calculator. UTI = urinary tract infection.

A flowchart for the management of 1000 simulated acutely ill children, assuming a UTI prevalence of 6%,^22^ 2%,^11^ or 8%^1^ are shown as Figure 4, and Supplementary Figures S2 and S3. The number of missed infections for each of the scores is 15/60 (UTICalc), 5/60 (Gorelick), and 55/60 (DUTY).

### Initiation of empirical treatment

When urine has been obtained based on the clinical model (*n* = 69), the UTICalc dipstick model had a sensitivity of 100% (95% CI = 29% to 100%) and specificity of 91% (95% CI = 79% to 97%), while the UTICalc ‘hemocytometer model’, including WBC/µl, had a sensitivity of 100% (95% CI = 29% to 100%) and specificity of 88% (95% CI = 75% to 95%). For the DUTY dipstick score ≥6 points, the sensitivity was 12% (95% CI = 2% to 30%) and specificity was 96% (95% CI = 93% to 98%).

Using the UTICalc dipstick model, no UTIs would be missed and 59% (*n* = 71/121 [95% CI = 49% to 68%]) of antibiotics would be given incorrectly (Figure 4), while using the dipstick test per standard care, one of three (*n* = 6/23) UTIs would be missed and 72% (*n* = 46/64 [95% CI = 59% to 82%]) of antibiotics would be given incorrectly (*P* = 0.1073). Using the DUTY dipstick score ≥6 points, few children would be tested, as the sensitivity of the clinical model was low. Therefore, the only child with a UTI would have been missed, and the only prescription given for UTI would have been incorrect. (Figure 4).

### Sensitivity analyses

Because the urine culture threshold is debatable in children,^23^ and to allow optimal between-rule comparison in this study, sensitivity analyses were performed comparing the diagnostic accuracies using identical criteria per rule.

At one pathogen ≥10^5^ CFU/ml,^6^ sensitivities and specificities were 71% (95% CI = 42% to 92%) and 28% (95% CI = 19% to 39%) for the UTICalc; 93% (95% CI = 68% to 100%) and 10% (95% CI = 5% to 17%) for the Gorelick score; and 8% (95% CI = 1% to 25%) and 99% (95% CI = 96% to 100%) for the DUTY score.

Using a lower threshold, for example, ≥5 × 10^4^ CFU/ml with pyuria,^15^ resulted in sensitivities and specificities of 75% (95% CI = 19% to 99%) and 16% (95% CI = 9% to 25%) for the UTICalc; 100% (95% CI = 40% to 100%) and 8% (95% CI = 4% to 16%) for the Gorelick score; and 12% (95% CI = 0% to 53%) and 98% (95% CI = 96% to 99%) for the DUTY score.

Because the Gorelick score was derived in girls, the sensitivity and specificity for girls were calculated separately (*n* = 38), which was 100% (95% CI = 48% to 100%) and 6% (95% CI = 1% to 20%).

## Discussion

### Summary

In the data, the sensitivities of the UTICalc (75%) and Gorelick score (91%) were moderate-to-high at low specificities (16% and 8%), leading to a very high number of children in whom a urine sample would have to be obtained (72% and 92%). This is much higher than the urine sampling rate per routine care measured in this study (38%). For the DUTY score, the sensitivity was low (8%) and the specificity was high (99%). The AUC showed little discriminatory value (0.55), meaning that urine sampling would only be done in 1% of children, but at the expense of missing the majority of UTIs.

When urine has been obtained, the UTICalc dipstick model appeared to be more sensitive than using the dipstick test as per routine care, based on very few cases.

### Strengths and limitations

This was a cross-sectional study in primary care with systematic urine sampling. Because of the pragmatic nature of this study, the results will likely reflect real-life clinical practice.

Caution is needed in the interpretation, because the sample size was low, and included only 26 children with UTI. The study was terminated early, at the start of the SARS-CoV-2 pandemic. Face-to-face clinical care was heavily restricted and GPs indicated that recruiting was no longer possible. Because the population was no longer representative of a normal spectrum of children seen in daily practice, it was decided to end study recruitment to obtain applicable results, but with much less precision. The calibration of the DUTY models was weak, most likely because there were too few UTI cases. Additionally, the original regression coefficients for the UTICalc and Gorelick score were not available, meaning that the models were assessed without recalibration.

It is possible that some children in whom UTI was suspected or urine collection was difficult were not included, owing to the need of an additional urine sample for clinical management. This may have caused selection bias and an underestimation of the sensitivity.

In a minority of cases (32%), urine samples were obtained using adhesive bags. Although adhesive bags result in a high amount of contamination, it was chosen to avoid using invasive procedures in order to obtain real-world results that are applicable to clinical practice. Misallocation bias owing to contaminated samples could have caused an underestimation of sensitivity and specificity (more false negatives and false positives).

Because the prediction rules were validated in primary care in Belgium, it is possible that the findings are not applicable to low-resource countries or ambulatory care settings where the population of children is different than the study’s case-mix. Because the target population that the original prediction rule was meant for was selected, this limits the generalisability of the findings.

### Comparison with existing literature

These results differ substantially from the derivation studies where sensitivities of 95%, 95%, and 52% were found for the UTICalc,^12^ Gorelick,^13^ and DUTY score,^11^ respectively.

The population of the DUTY study was most comparable with the target population; however, the score’s sensitivity in the study was lower. Possible reasons are the low sample size of the study, over-fitting in the original study, selection bias in the current data, or random error.

The specificities of the UTICalc and Gorelick score were substantially lower than in the original studies. This might have been caused by differences in study design (retrospective derivation),^12^ or selection bias in the data. Additionally, the case-mix of children in these studies included a more severe spectrum in whom UTI was suspected,^12,13^ the population was more diverse (25% Black),^12^ and there was a higher circumcision rate (19%).^12^

The urine sampling rate per normal care in this study (32%–38%) was higher than in other studies performed in general practices,^11^ which could have been caused by selection bias, a Hawthorne effect owing to the nature of the study, or the availability of a study-specific urine sample, per protocol.

### Implications for practice

If future external validation and impact analysis confirm the findings, the UTICalc or Gorelick score could be useful to decrease the number of missed UTIs in children in general practice. These simple metrics could be easily implemented to determine which children require urine sampling. One advantage of the UTICalc is its integration of urine dipstick test results and, therefore, this score might be useful to avoid excessive use of urine culture, if proven sensitive.

Novel point-of-care tests for UTI should be assessed in combination with a clinical prediction rule before implementation because clinical suspicion of UTI is not sensitive enough to identify children for testing.

## Availability of data and material:

The data underlying this article will be shared on reasonable request to the corresponding author.

## Trial registration number

The ERNIE4 study was pre-registered (clinicaltrials.gov: NCT03835104).
